# Electronic Properties of Möbius Cyclacenes Studied by Thermally-Assisted-Occupation Density Functional Theory

**DOI:** 10.1038/s41598-019-39524-4

**Published:** 2019-02-27

**Authors:** Jui-Hui Chung, Jeng-Da Chai

**Affiliations:** 1 Department of Physics, National Taiwan University, Taipei, 10617 Taiwan; 2 Center for Theoretical Physics, National Taiwan University, Taipei, 10617 Taiwan; 3 Center for Quantum Science and Engineering, National Taiwan University, Taipei, 10617 Taiwan

## Abstract

It has been extremely difficult for traditional theoretical methods to adequately predict the properties of systems possessing radical character (i.e., multi-reference systems), especially for multi-reference systems at the nanoscale. To circumvent this, we employ thermally-assisted-occupation density functional theory (TAO-DFT) to predict the electronic properties of Möbius cyclacenes, with the number of fused benzene rings (*n*) ranging from 8 to 100. In addition, to investigate the significance of Möbius topology, we also compare these properties with the respective properties of cyclacenes and acenes, containing the same number of fused benzene rings. From our TAO-DFT results, Möbius cyclacenes, cyclacenes, and acenes have singlet ground states for all the cases examined. However, unlike acenes, the electronic properties of Möbius cyclacenes and cyclacenes display clear oscillation patterns when *n* is small (e.g., *n* ≤ 10 for Möbius cyclacenes and *n* ≤ 23 for cyclacenes), and converge to the respective properties of acenes when *n* greatly exceeds 30. The polyradical character of the ground states of Möbius cyclacenes should increase with the molecular size, intimately correlated with the localization of active orbitals at the edges of molecules.

## Introduction

Recently, graphene nanoribbons (GNRs) have emerged as promising quasi-one-dimensional (Q1D) materials for next-generation electronic nanodevices^1–7^. Because of the effect of edges and quantum confinement, GNRs can exhibit band gaps for transistor operation with exceptional switching speed and high carrier mobility. However, the properties of GNRs are intimately correlated with the geometrical arrangements of GNRs; they are rendered into semimetal without band gaps when widening into their two-dimensional parent material, graphene. Particularly, GNRs with zigzag edges (ZGNRs) are expected to host edge-localized states, which may serve as key elements for graphene-based electronics and spintronics. Therefore, a comprehensive structural analysis of GNRs is of fundamental importance for building high-performance GNR-based nanodevices.

While the local geometries of nanomaterials have profound consequences on their properties, such as the surface states affecting the bulk crystals, and the edge states affecting graphene, the global topology of Möbius graphene nanoribbons (MGNRs), with non-orientable global invariants, can significantly influence their electronic properties. Note that a MGNR can be constructed by joining the two ends of a ZGNR with a single half-twist. Accordingly, a MGNR has only one edge and one surface. Due to their intriguing topologies, MGNRs have attracted extensive attention from the research community. Theoretical predictions of their electronic^8–10^, magnetic^11,12^, transport^13^, and thermal^14,15^ properties have been made in recent years. Furthermore, MGNRs were predicted to behave as topological insulators^16,17^. Whereas the conducting edge states of ZGNRs are very fragile, for systems with non-trivial topology, such as MGNRs, the Hamiltonians for the edge states are invariant to small perturbations, bringing tremendous possibilities to realize topological insulators^18^.

**Figure 1:**
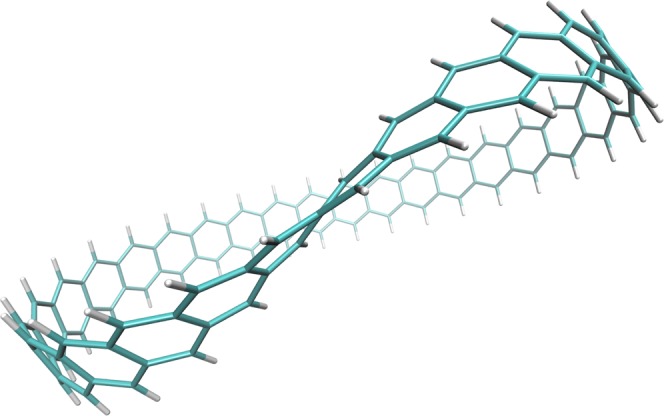
Structure of Möbius 30-cyclacene, which contains 30 fused benzene rings forming a closed loop with a single half-twist.

In particular, the properties of the fundamental repeating units of MGNRs may require further investigation. In the present work, our investigation focuses on a series of Möbius *n*-cyclacenes (see Fig. 1). Note that Möbius *n*-cyclacene can be constructed by joining the two ends of *n*-acene (i.e., a Q1D molecule containing *n* fused benzene rings^19,20^) with a single half-twist. Möbius *n*-cyclacenes, which belong to the category of aromatic hydrocarbons, are delocalized *π*-conjugated systems. Aromaticity is a key concept in chemistry in that aromatic molecules display enhanced chemical stability and induced aromatic ring currents^21^. The peculiar chemical properties of aromatics are amplified by Möbius topologies, where the topics of Möbius aromaticity have recently attracted considerable interest^22,23^. Aromaticity is characterized by the number of delocalized *π*-electrons. For the untwisted case, i.e., *n*-cyclacene (as shown in Fig. 1 of ref.^24^), there are two annulene peripheral circuits joined by transannular bonds to form *n* fused benzene rings. The annulene periphery, following Hückel’s rule, is only stable (aromatic), when the number of *π*-electrons is 4*k* + 2, with *k* being an integer (corresponding to an odd number of benzene rings). Möbius *n*-cyclacene, however, has only one edge and thus only one annulene periphery of twofold the size as the comparing the untwisted case. Therefore, the single annulene periphery forming Möbius *n*-cyclacene always includes 4*k π*-electrons, irrespective of the number of benzene rings, which violates Hückel’s rule. However, Zimmerman^25^ and Heilbronner^26^ argued that for the Möbius topology, these molecules rather need 4*k π*-electrons to achieve aromaticity and stability. The single half-twist of the structure was concluded to be correlated with the interesting properties of Möbius *n*-cyclacenes.

Note that the Möbius strip was first discovered by August Ferdinand Möbius (a German mathematician) in 1858. While the first prediction on the possibility of forming Möbius aromaticity was made in 1964^26^, the first stable Möbius aromatic hydrocarbon was synthesized only very recently^27^. Although the prospects of successful synthesis of Möbius cyclacenes may not be ideal^28^, progress has been made in synthesizing different kinds of microscopic Möbius strips: the Möbius structure formed by NbSe_2_ crystals was obtained under unconventional growth conditions^29^; the design and synthesis of the first triply twisted Möbius annulene was recently proposed^30^. Besides, twisted GNRs were obtained inside carbon nanotubes (CNTs), offering the possibility of building MGNRs^31^. Recently, the successful on-surface generation of triangulene was reported^32^. Note that triangulene, consisting of six fused benzene rings, belongs to the category of topologically nontrivial structures, such as Möbius *n*-cyclacenes. Studying Möbius *n*-cyclacenes may be crucial for the atomically controlled bottom-up fabrication of MGNRs as well. Therefore, a comprehensive study of the properties of Möbius *n*-cyclacenes is of essential importance. These properties are expected to be constructive for probing the potential applications of their quantum effects induced by the nontrivial topological configurations.

As of now, the studies of Möbius *n*-cyclacenes have mainly been performed theoretically; yet it remains rather difficult to make reliable prediction on the electronic properties of the larger Möbius *n*-cyclacenes, possibly due to their *π*-conjugation and polyradical character. Note that Kohn-Sham density functional theory (KS-DFT)^33^ employing traditional (semilocal^10,11^ and hybrid^34–36^) exchange-correlation (XC) functionals may not reliably predict the properties of multi-reference (MR) systems (i.e., systems possessing radical character)^37–39^. Note that *π*-conjugated polyradical systems usually require *ab initio* MR electronic structure methods^19,20,40–42^. However, calculations based on *ab initio* MR electronic structure methods are computationally infeasible for MR systems at the nanoscale (especially for geometry relaxation). Accordingly, it remains extremely difficult for traditional theoretical methods to adequately predict the properties of the larger Möbius *n*-cyclacenes.

To achieve a favorable balance between cost and performance for studying MR systems at the nanoscale, thermally-assisted-occupation density functional theory (TAO-DFT)^43^ has recently been proposed. From the physical statements provided in Section III.E of ref.^43^ and the numerical results given in Section IV of ref.^43^, the static correlation energy of a system can be adequately described by the entropy contribution (which can be expressed by the fictitious temperature (*θ*) and orbital occupation numbers in TAO-DFT), even for TAO-DFT employing a local XC density functional. Just like the static correlation energy of a system, the entropy contribution in TAO-DFT, which is always nonpositive, is negligible for a single-reference (SR) system (i.e., a system possessing non-radical character), and can significantly lower the total energy of a MR system. Accordingly, TAO-DFT reduces to KS-DFT for SR systems, and outperforms KS-DFT for MR systems. Existing semilocal and hybrid XC functionals in KS-DFT may be employed in TAO-DFT as well^44,45^. Very recently, a self-consistent scheme for determining the fictitious temperature in TAO-DFT has also been proposed for a diverse range of applications^46^.

Since TAO-DFT is similar to KS-DFT in computational efficiency, TAO-DFT has been widely applied to study the electronic properties of various MR systems at the nanoscale^24,47–52^. Therefore, in the present study, we adopt TAO-DFT to investigate the electronic properties of Möbius *n*-cyclacenes (*n* = 8–100). Besides, to assess the significance of Möbius topology, we also compare the electronic properties of Möbius *n*-cyclacenes with the respective properties of *n*-cyclacenes^24,53,54^ and *n*-acenes^19,20,43,44,47,55^.

## Computational Details

We perform all calculations with Q-Chem 4.3^56^, adopting the 6–31 G(d) basis set and the numerical grid containing 75 radial points in the Euler-Maclaurin quadrature and 302 angular points in the Lebedev grid. Results are obtained from TAO-LDA^43^ (TAO-DFT adopting the local density approximation (LDA) XC functional^57,58^ and the LDA *θ*-dependent functional) with *θ* = 7 mhartree.

Here, we briefly explain the reason that *θ* = 7 mhartree is chosen. In our previous study^43^, TAO-LDA (with some fictitious temperature *θ*) has been shown to perform reasonably well for MR systems, providing that the corresponding orbital occupation numbers are close to the natural orbital occupation numbers (NOONs). In such a situation, the strong static correlation effects can be adequately described by the entropy contribution of TAO-LDA. However, this implies that a *θ* related to the NOONs should be adopted. For simplicity and computational efficiency, TAO-LDA with a system-independent *θ* is favorable. Accordingly, in our previous study^43^, the optimal *θ* value has been defined as the largest *θ* value for which TAO-LDA performs similarly to KS-LDA (i.e., KS-DFT with the LDA XC functional, which is TAO-LDA with *θ* = 0) for SR systems, yielding an optimal *θ* = 7 mhartree based on the numerical investigations. TAO-LDA (*θ* = 7 mhartree), though not optimal for all systems, has been shown to consistently improve upon KS-LDA for MR systems, while performing similarly to KS-LDA for SR systems. Besides, in a recent study^48^, the orbital occupation numbers obtained from TAO-LDA (*θ* = 7 mhartree) have been shown to be qualitatively similar to the NOONs obtained from the active-space variational two-electron reduced-density-matrix (RDM-CASSCF) method^41^ (i.e., an accurate MR electronic structure method), yielding a similar trend for the radical character of the 24 alternant PAHs (polycyclic aromatic hydrocarbons) studied. Due to its computational efficiency and reasonable accuracy, we adopt TAO-LDA (*θ* = 7 mhartree) in the present study.

Note also that TAO-DFT has been extended to the generalized-gradient approximation (GGA) XC functionals^44^. However, in TAO-DFT, the GGAs improve upon the LDA mainly for the properties governed by short-range XC effects, not for the properties governed by strong static correlation effects. As shown in our previous study^44^, in TAO-DFT, the GGAs have similar performance as the LDA for the electronic properties of *n*-acenes (i.e., systems with strong static correlation effects). Since *n*-cyclacene can be regarded as an interconnection of *n*-acene, and Möbius *n*-cyclacene can be regarded as *n*-cyclacene with a single half-twist, we expect that the electronic properties of Möbius *n*-cyclacene/*n*-cyclacene/*n*-acene obtained with the LDA and GGAs in TAO-DFT should remain similar, especially for very large *n*.

## Results and Discussion

### Singlet-Triplet Energy Gap

To determine the ground state of Möbius *n*-cyclacene/*n*-cyclacene/*n*-acene (*n* = 8–100), we perform calculations based on spin-unrestricted TAO-LDA to obtain the lowest singlet and lowest triplet states of Möbius *n*-cyclacene/*n*-cyclacene/*n*-acene, with the corresponding geometries being completely relaxed. Subsequently, we calculate the singlet-triplet energy gap of Möbius *n*-cyclacene/*n*-cyclacene/*n*-acene as

1 $${E}_{{\rm{ST}}}={E}_{{\rm{T}}}-{E}_{{\rm{S}}},$$

with *E*_T_ and *E*_S_ being the lowest triplet and lowest singlet energies, respectively, of Möbius *n*-cyclacene/*n*-cyclacene/*n*-acene.

**Figure 2:**
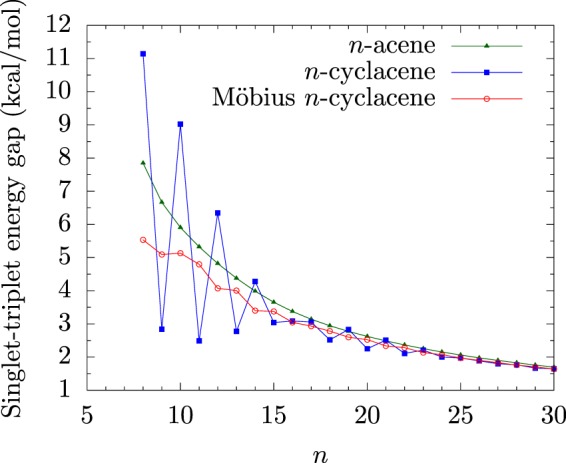
Singlet-triplet energy gap of Möbius *n*-cyclacene/*n*-cyclacene/*n*-acene (*n* = 8–30), obtained with spin-unrestricted TAO-LDA.

**Figure 3:**
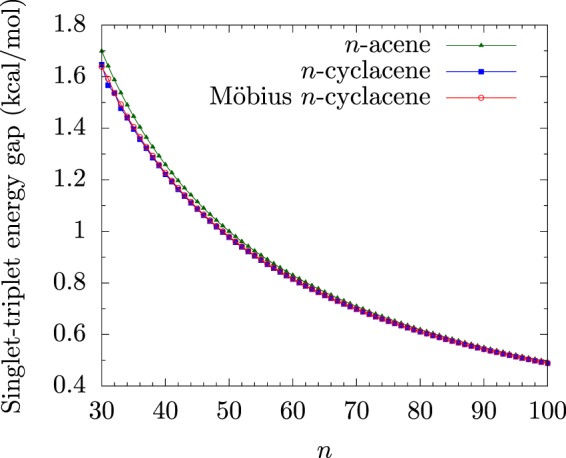
Singlet-triplet energy gap of Möbius *n*-cyclacene/*n*-cyclacene/*n*-acene (*n* = 30–100), obtained with spin-unrestricted TAO-LDA.

From our results (see Figs 2 and 3), Möbius *n*-cyclacenes, *n*-cyclacenes, and *n*-acenes have singlet ground states for all the cases studied (i.e., *n* = 8–100). As *n* increases, the *E*_ST_ value of *n*-acene decreases monotonically. By contrast, the smaller Möbius *n*-cyclacenes (e.g., *n* ≤ 10) and the smaller *n*-cyclacenes (e.g., *n* ≤ 23) exhibit cryptoannulenic effects^8^, displaying oscillatory patterns in the respective *E*_ST_ values. Based on the oscillation amplitudes of the *E*_ST_ values, *n*-cyclacenes should exhibit more prominent cryptoannulenic effects than Möbius *n*-cyclacenes. When *n* greatly exceeds 30, the *E*_ST_ values of Möbius *n*-cyclacene and *n*-cyclacene monotonically converge from below to the *E*_ST_ value of *n*-acene (see Supplementary Information (SI), Table S1 for details).

As mentioned previously, for *π*-conjugated systems, such as Möbius *n*-cyclacenes, the predictions from KS-DFT employing traditional XC density functional can be problematic^37–39^. For example, Möbius *n*-cyclacenes (*n* = 8–20) were previously predicted to have triplet ground states, based on KS-B3LYP (the B3LYP hybrid functional in KS-DFT)^36^. The contradictory results obtained from KS-B3LYP can be artifacts, intimately correlated with spin contamination (i.e., the artificial mixing of different electronic spin-states)^10,11,24,36,43,44,47,52,53,59,60^. Note that spin contamination is not a systematic error, and hence, the energy difference between spin-states can be adversely influenced.

Because of the symmetry constraint^24,43,44,47,55^, for the exact theory, the lowest singlet energy of Möbius *n*-cyclacene/*n*-cyclacene/*n*-acene obtained from spin-restricted calculations should be the same as the corresponding energy obtained from spin-unrestricted calculations. However, this condition may not be satisfied by KS-DFT with traditional XC functionals (because of the spin contamination mentioned above), as shown in recent studies on Möbius *n*-cyclacenes^10,11,36^, *n*-cyclacenes^24,53^, and *n*-acenes^19,20,43,44,47,55^. To assess whether this condition can be satisfied by TAO-LDA, we additionally perform calculations based on spin-restricted TAO-LDA to obtain the lowest singlet states of Möbius *n*-cyclacenes, *n*-cyclacenes, and *n*-acenes, with the corresponding geometries being fully relaxed. The lowest singlet energy of Möbius *n*-cyclacene/*n*-cyclacene/*n*-acene obtained from spin-restricted TAO-LDA is found to be numerically identical to the corresponding energy obtained from spin-unrestricted TAO-LDA, implying that our calculations based on spin-unrestricted TAO-LDA do not lead to unphysical symmetry-breaking solutions.

### Vertical Ionization Potential, Vertical Electron Affinity, and Fundamental Gap

Here, we investigate the possibility of Möbius *n*-cyclacene/*n*-cyclacene/*n*-acene for photovoltaic applications. At the completely relaxed geometry of the ground state (i.e., the lowest singlet state) of Möbius *n*-cyclacene/*n*-cyclacene/*n*-acene, we perform calculations based on spin-unrestricted TAO-LDA to obtain the vertical ionization potential

2 $${{\rm{IP}}}_{v}={E}_{total}({\rm{cation}})-{E}_{total}({\rm{neutral}}),$$

vertical electron affinity

3 $${{\rm{EA}}}_{v}={E}_{total}({\rm{neutral}})-{E}_{total}({\rm{anion}}),$$

and fundamental gap

4 $${E}_{g}={{\rm{IP}}}_{v}-{{\rm{EA}}}_{v},$$

where *E*_*total*_(neutral), *E*_*total*_(cation), and *E*_*total*_(anion) are the total energies of the neutral, cationic, and anionic states.

**Figure 4:**
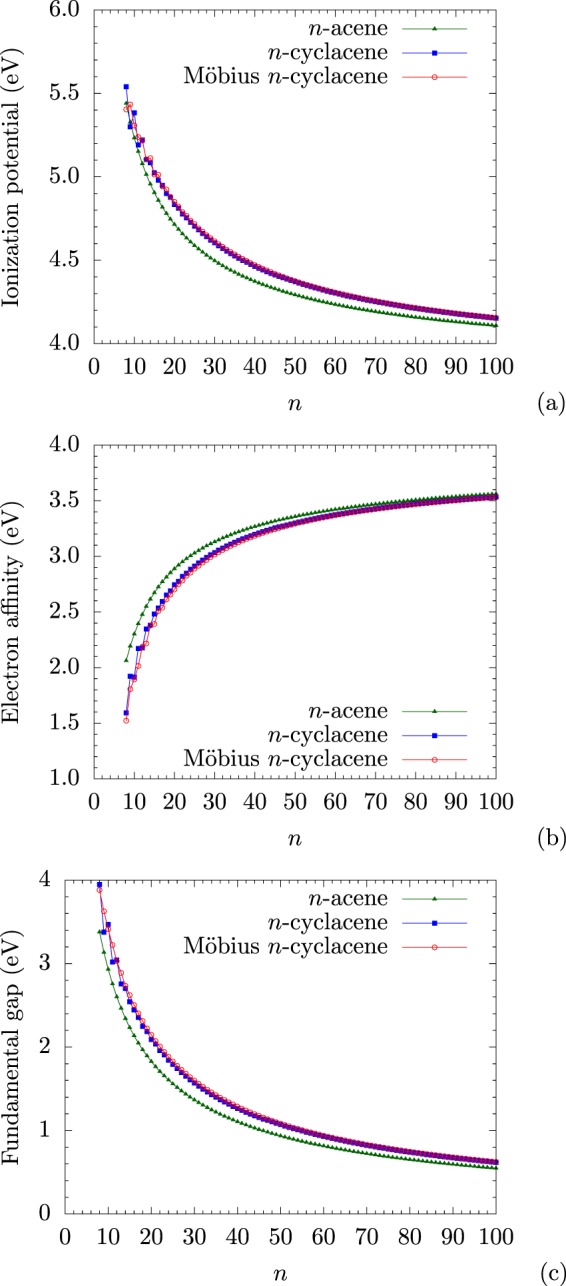
(**a**) Vertical ionization potential, (**b**) vertical electron affinity, and (**c**) fundamental gap for the ground state of Möbius *n*-cyclacene/*n*-cyclacene/*n*-acene, obtained with spin-unrestricted TAO-LDA.

As shown in Fig. 4, with the increase of *n*, the IP_*v*_ value of *n*-acene decreases monotonically, the EA_*v*_ value of *n*-acene increases monotonically, and hence, the *E*_*g*_ value of *n*-acene decreases monotonically. By contrast, as *n* increases, the IP_*v*_ values of Möbius *n*-cyclacene and *n*-cyclacene decrease with oscillatory patterns, and the EA_*v*_ values of Möbius *n*-cyclacene and *n*-cyclacene increase with oscillatory patterns. Note that the oscillation amplitudes of the IP_*v*_ and EA_*v*_ values of *n*-cyclacenes are larger than those of Möbius *n*-cyclacenes, implying that *n*-cyclacenes should possess more prominent cryptoannulenic effects than Möbius *n*-cyclacenes. Besides, with increasing *n*, these oscillatory patterns reduce gradually, and disappear eventually. When *n* greatly exceeds 30, the IP_*v*_ values of Möbius *n*-cyclacene and *n*-cyclacene monotonically converge from above to the IP_*v*_ value of *n*-acene, the EA_*v*_ values of Möbius *n*-cyclacene and *n*-cyclacene monotonically converge from below to the EA_*v*_ value of *n*-acene, and the *E*_*g*_ values of Möbius *n*-cyclacene and *n*-cyclacene monotonically converge from above to the *E*_*g*_ value of *n*-acene (see SI, Tables S2 to S4 for details). Particularly, the *E*_*g*_ value of Möbius *n*-cyclacene (*n* = 13–55) is between 1 and 3 eV (i.e., the desirable regime for photovoltaic applications).

### Symmetrized von Neumann Entropy

Here, we examine the potential polyradical character of Möbius *n*-cyclacene/*n*-cyclacene/*n*-acene by calculating the symmetrized von Neumann entropy^24,44,47,55^

5 $${S}_{{\rm{vN}}}=-\,\frac{1}{2}\,\sum _{\sigma =\alpha ,\beta }\,\sum _{i=1}^{\infty }\,\{{f}_{i,\sigma }\,\mathrm{ln}({f}_{i,\sigma })+(1-{f}_{i,\sigma })\,\mathrm{ln}(1-{f}_{i,\sigma })\}$$

for the ground state of Möbius *n*-cyclacene/*n*-cyclacene/*n*-acene using spin-unrestricted TAO-LDA. In Eq. (5), *f*_*i*,*σ*_ (i.e., the occupation number of the *i*^th^ *σ*-spin orbital (*σ* = *α* or *β*) calculated using spin-unrestricted TAO-LDA), which takes a value between zero and one, is close to the occupation number of the *i*^th^ *σ*-spin natural orbital^43–45,48^. For a SR system ({*f*_*i*,*σ*_} are approximately equal to either zero or one), *S*_vN_ is very small. Nevertheless, for a MR system ({*f*_*i*,*σ*_} are very different from either zero or one for active spin-orbitals (i.e., fractionally occupied spin-orbitals), and are approximately equal to either zero or one for others), *S*_vN_ increases with the number of fractionally occupied spin-orbitals.

**Figure 5:**
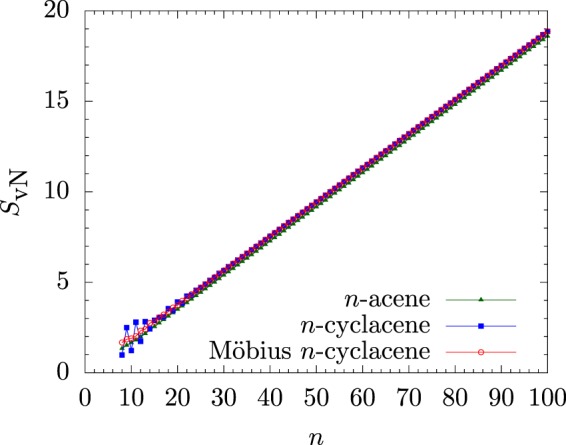
Symmetrized von Neumann entropy (*S*_vN_) for the ground state of Möbius *n*-cyclacene/*n*-cyclacene/*n*-acene, obtained with spin-unrestricted TAO-LDA.

As presented in Fig. 5, the *S*_vN_ value of *n*-acene increases monotonically with the molecular size. By contrast, with the increase of *n*, the *S*_vN_ values of Möbius *n*-cyclacene and *n*-cyclacene increase with oscillatory patterns. However, these oscillatory patterns reduce gradually, and disappear eventually, as *n* increases. When *n* greatly exceeds 30, the *S*_vN_ values of Möbius *n*-cyclacene and *n*-cyclacene monotonically converge from above to the *S*_vN_ value of *n*-acene (see SI, Table S5 for details). Accordingly, just like previous findings for *n*-cyclacenes^24,54^ and *n*-acenes^19,20,43–45,47,55^, the polyradical character of the ground states of Möbius *n*-cyclacenes should increase with the molecular size.

### Occupation Numbers of Active Orbitals

**Figure 6:**
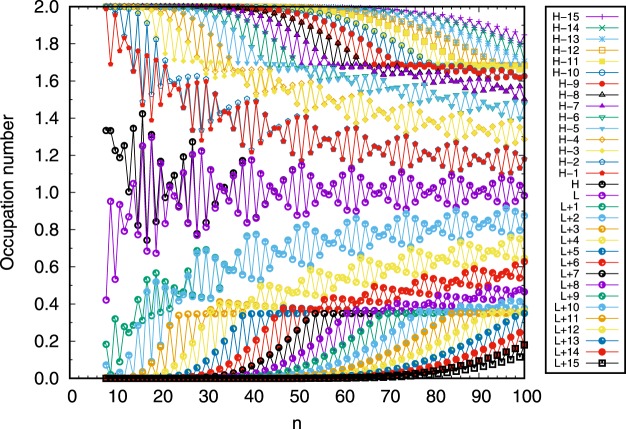
Occupation numbers of active orbitals for the ground state of Möbius *n*-cyclacene, obtained with spin-restricted TAO-LDA. Here, for simplicity, HOMO and LUMO are referred to as H and L, respectively.

The occupation numbers of active orbitals in TAO-DFT provide valuable information for directly assessing the polyradical character of Möbius *n*-cyclacene^43–45,48^. To further demonstrate the reasons of the increase of *S*_vN_ with the size of molecule, the occupation numbers of active orbitals for the ground state of Möbius *n*-cyclacene, obtained with spin-restricted TAO-LDA, are plotted in Fig. 6. For Möbius *n*-cyclacene (with *N* electrons), the highest occupied molecular orbital (i.e., the (*N*/2)^th^ orbital) and lowest unoccupied molecular orbital (i.e., the (*N*/2 + 1)^th^ orbital) are referred to as the HOMO and LUMO, respectively^24,43,47^. As shown, with the increase of *n*, more and more orbitals have an occupation number close to one (i.e., more and more spin-orbitals have an occupation number close to 0.5), supporting that the polyradical character of the ground states of Möbius *n*-cyclacenes should increase with the molecular size.

### Visualization of Active Orbitals

**Figure 7:**
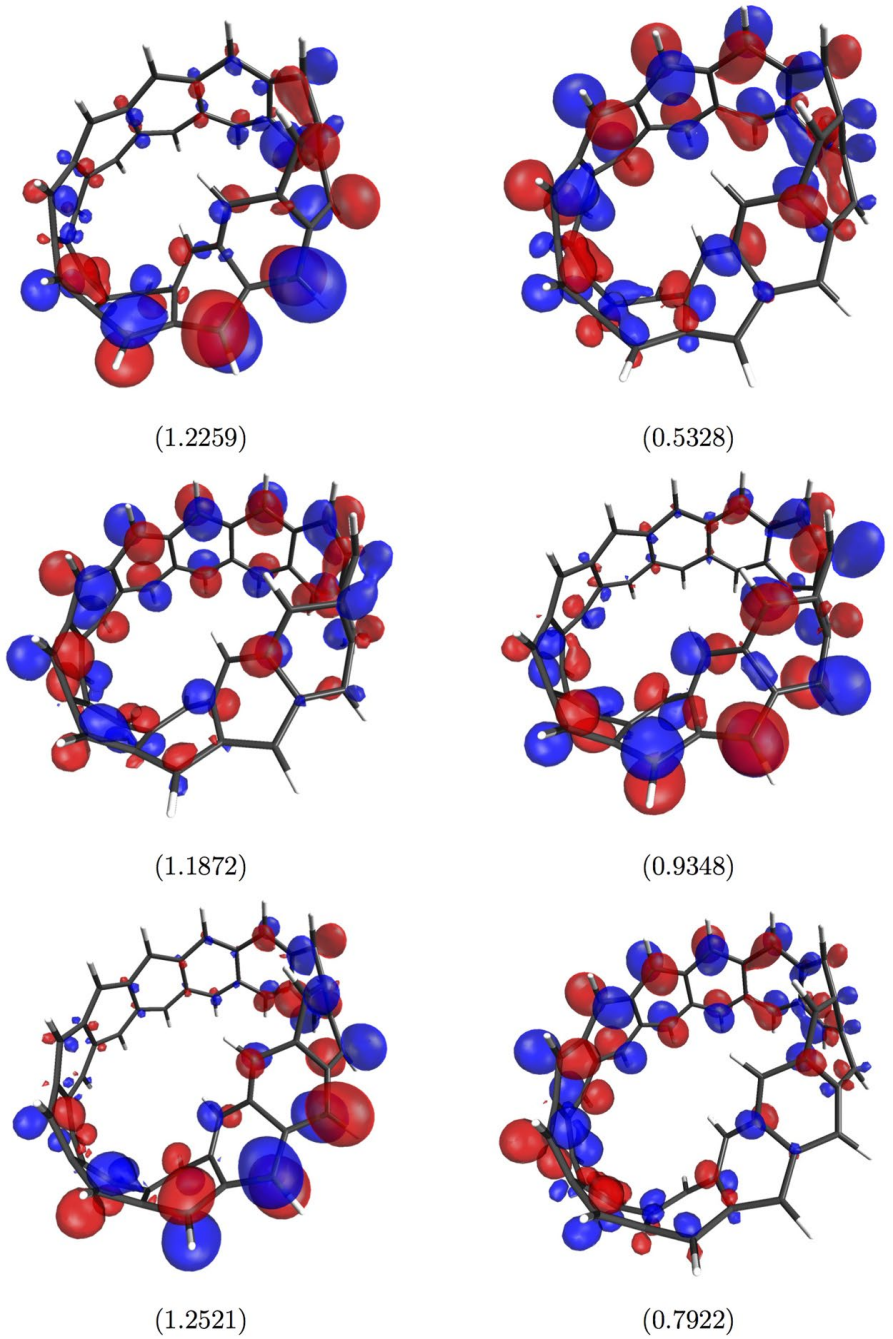
Visualization of the HOMO (left-hand side) and LUMO (right-hand side) for the ground state of Möbius 10-cyclacene (top)/Möbius 11-cyclacene (middle)/Möbius 12-cyclacene (bottom), obtained with spin-restricted TAO-LDA. Here, the isovalue is 0.03 e/Å^3^, and the occupation numbers of HOMOs and LUMOs are shown in parentheses.

**Figure 8:**
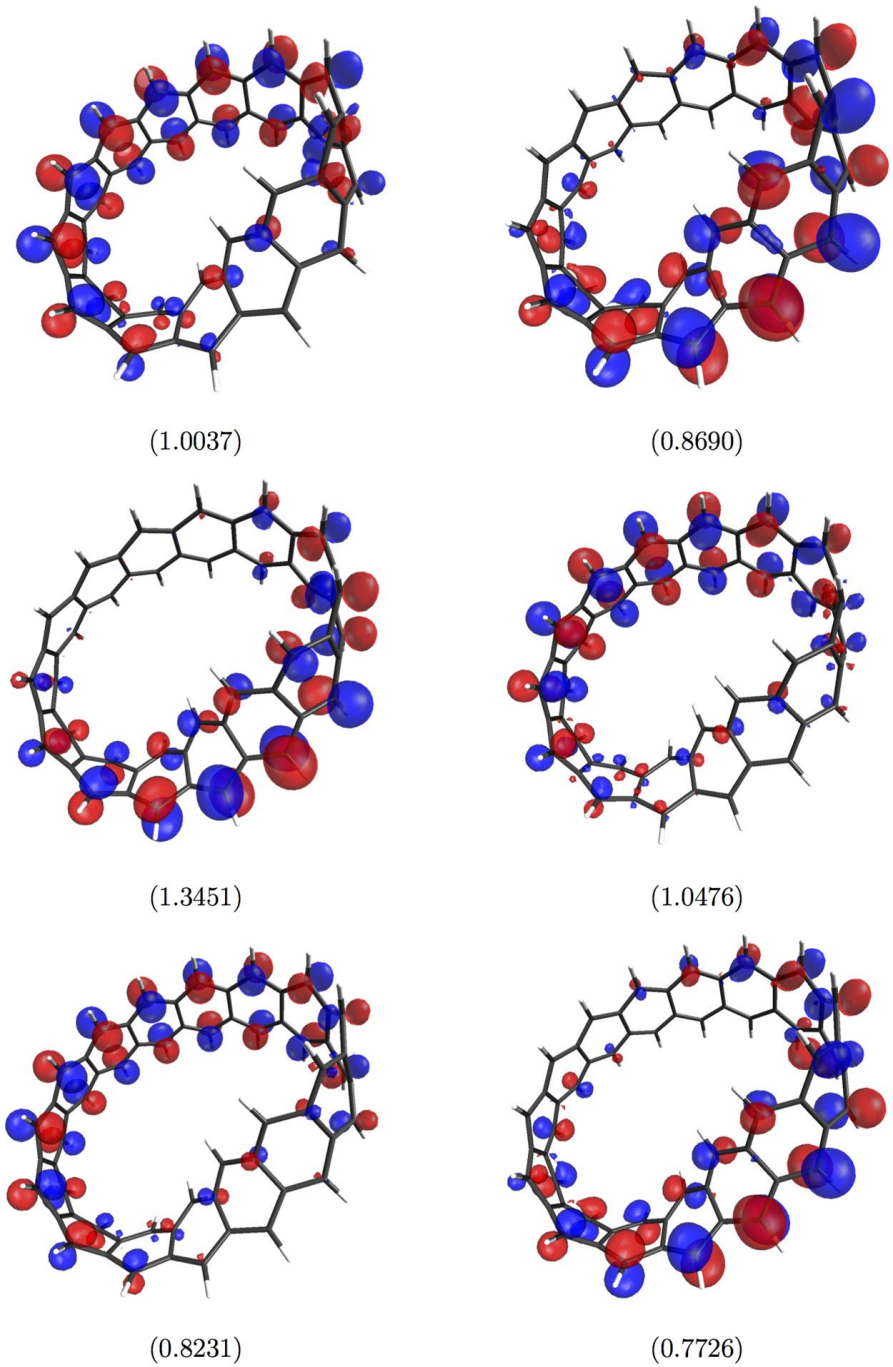
Visualization of the HOMO (left-hand side) and LUMO (right-hand side) for the ground state of Möbius 13-cyclacene (top)/Möbius 14-cyclacene (middle)/Möbius 15-cyclacene (bottom), obtained with spin-restricted TAO-LDA. Here, the isovalue is 0.03 e/Å^3^, and the occupation numbers of HOMOs and LUMOs are shown in parentheses.

The active orbitals, such as HOMOs and LUMOs, for the ground states of a few illustrative Möbius *n*-cyclacenes (*n* = 10–15), calculated using spin-restricted TAO-LDA, are plotted in Figs 7 and 8. As shown, the active orbitals are mostly concentrated at the edges of Möbius *n*-cyclacenes. Note that the visualization of active orbitals for the ground states of a few illustrative *n*-acenes (as presented in Fig. 14 of ref.^47^) and *n*-cyclacenes (as presented in Fig. 10 of ref.^24^), obtained with spin-restricted TAO-LDA, can be found in previous studies for comparison. Just like previous findings for *n*-cyclacenes^24,54^ and *n*-acenes^19,20,47,55^, the increasing polyradical character of the larger Möbius *n*-cyclacenes should be intimately related to the localization of active orbitals at the edges of molecules, clearly increasing with the increase of *n*.

### Ground-State Geometry

There is a close relationship between curvature and topology in nanocarbon systems^61^. To investigate the effect of the ground-state geometry of Möbius *n*-cyclacene on the electronic properties, we examine the Gaussian curvature of Möbius *n*-cyclacene. Note that since *n*-acene possesses a planar geometry, and *n*-cyclacene possesses a cylindrical geometry, the Gaussian curvature of *n*-acene/*n*-cyclacene is zero everywhere.

**Figure 9:**
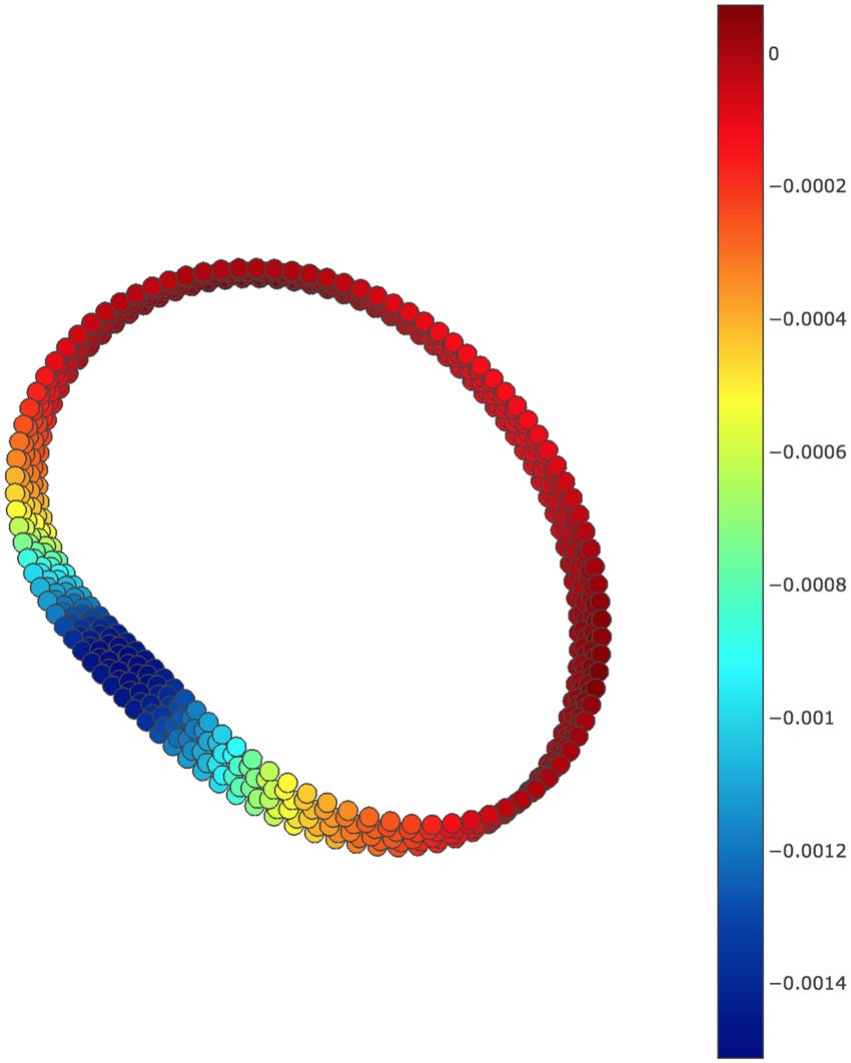
Ground-state geometry of Möbius 100-cyclacene (color coded with the value of Gaussian curvature (Å^−2^)), obtained with spin-unrestricted TAO-LDA.

In the present study, we adopt the initial starting geometry of Möbius *n*-cyclacene given by the one with a twist evenly distributed along the whole chain^28^, where the length and width of the chain are taken from the length and width, respectively, of *n*-acene. After full relaxation, the ground-state geometry of Möbius *n*-cyclacene (with *n* = 100 as an illustrative example), colored by Gaussian curvature, obtained with spin-unrestricted TAO-LDA, is plotted. Here, to determine the discrete approximation to the Gaussian curvature, we employ a local least squares surface fit of cubic polynomials for *k*-nearest neighbors (with *k* = 50). As shown in Fig. 9, the ground-state (i.e., energetically preferred) geometry of Möbius *n*-cyclacene is composed mostly of an essentially untwisted open chain (see the red region with zero Gaussian curvature) plus a highly twisted stripe (see the blue region with negative Gaussian curvature), i.e., the twist is not evenly distributed along the whole chain.

## Conclusions

In summary, the electronic properties (e.g., *E*_ST_, IP_*v*_, EA_*v*_, *E*_*g*_, *S*_vN_, and the occupation numbers and visualization of active orbitals) of Möbius *n*-cyclacenes (*n* = 8–100) have been studied using the recently proposed TAO-DFT, because of its computational efficiency and reasonable accuracy for the study of MR systems at the nanoscale. Since the ground states of the larger Möbius *n*-cyclacenes have been shown to possess increasing polyradical character, calculations based on traditional XC functionals in KS-DFT may not adequately predict the properties of Möbius *n*-cyclacenes, and calculations based on *ab initio* MR electronic structure methods are computationally intractable for MR systems at the nanoscale (e.g., the larger Möbius *n*-cyclacenes). Consequently, the use of TAO-DFT in the present study is well justified. Based on our TAO-DFT results, the larger Möbius *n*-cyclacenes, which have the smaller *E*_ST_ values, smaller *E*_*g*_ values, larger *S*_vN_ values, and more pronounced polyradical character, should exhibit stronger static correlation effects than the smaller Möbius *n*-cyclacenes.

To examine the significance of Möbius topology, we have also compared the electronic properties of Möbius *n*-cyclacenes with the respective properties of *n*-cyclacenes and *n*-acenes. Möbius *n*-cyclacenes, *n*-cyclacenes, and *n*-acenes have singlet ground states for all the cases studied (i.e., *n* = 8–100). However, unlike *n*-acenes, the electronic properties of Möbius *n*-cyclacenes and *n*-cyclacenes reveal oscillatory patterns when *n* is small (e.g., *n* ≤ 10 for Möbius *n*-cyclacenes and *n* ≤ 23 for *n*-cyclacenes), and converge to the respective properties of *n*-acenes when *n* greatly exceeds 30. These oscillatory patterns should be intimately correlated with the cryptoannulenic effects of Möbius *n*-cyclacenes and *n*-cyclacenes, which have been found to be significant only for the smaller *n*. Just like previous findings for *n*-cyclacenes and *n*-acenes, the increasing polyradical character of the larger Möbius *n*-cyclacenes should be intimately related to the localization of active orbitals at the edges of molecules, clearly increasing with the increase of *n*.

## Supplementary information

Supplementary Material to: Electronic Properties of Möbius Cyclacenes Studied by Thermally-Assisted-Occupation Density Functional Theory

## References

[1] Fujita M, Wakabayashi K, Nakada K, Kusakabe K (1996). Peculiar localized state at zigzag graphite edge. J. Phys. Soc. Jpn..

[2] Nakada K, Fujita M, Dresselhaus G, Dresselhaus MS (1996). Edge state in graphene ribbons: nanometer size effect and edge shape dependence. Phys. Rev. B.

[3] Wakabayashi K, Fujita M, Ajiki H, Sigrist M (1999). Electronic and magnetic properties of nanographite ribbons. Phys. Rev. B.

[4] Barone V, Hod O, Scuseria GE (2006). Electronic structure and stability of semiconducting graphene nanoribbons. Nano Lett..

[5] Ritter KA, Lyding JW (2009). The influence of edge structure on the electronic properties of graphene quantum dots and nanoribbons. Nat. Mater..

[6] Han W, Kawakami RK, Gmitra M, Fabian J (2014). Graphene spintronics. Nat. Nanotechnol..

[7] Ruffieux P (2016). On-surface synthesis of graphene nanoribbons with zigzag edge topology. Nature.

[8] Türker L (1998). MNDO treatment of the Hückel and Möbius types of cyclacenes. J. Molecular Structure.

[9] Yamashiro A, Shimoi Y, Harigaya K, Wakabayashi K (2004). Novel electronic states in graphene ribbons-competing spin and charge orders. Physica E.

[10] Wang X, Zheng X, Ni M, Zou L, Zeng Z (2010). Theoretical investigation of Möbius strips formed from graphene. Appl. Phys. Lett..

[11] Jiang D-E, Dai S (2008). Spin states of zigzag-edged Möbius graphene nanoribbons from first principles. J. Phys. Chem. C.

[12] Wakabayashi K, Harigaya K (2003). Magnetic structure of nano-graphite Möbius ribbon. J. Phys. Soc. Jpn..

[13] Takaki H, Kobayashi N (2011). Quantum transport properties of zigzag graphene nanoribbons. Physica E.

[14] Jiang J-W, Wang J-S, Li B (2010). Topological effect on thermal conductivity in graphene. J. Appl. Phys..

[15] Jiang JW, Wang JS, Li B (2010). Topology-induced thermal rectification in carbon nanodevice. EPL.

[16] Guo ZL, Gong ZR, Dong H, Sun CP (2009). Möbius graphene strip as a topological insulator. Phys. Rev. B.

[17] Gong Z-R, Song Z, Sun C-P (2016). Quasi-one dimensional topological insulator: Möbius molecular devices in Peierls transition. Commun. Theor. Phys..

[18] Zhang S-C (2008). Topological states of quantum matter. Physics.

[19] Hachmann J, Dorando JJ, Avilés M, Chan GK-L (2007). The radical character of the acenes: a density matrix renormalization group study. J. Chem. Phys..

[20] Mizukami W, Kurashige Y, Yanai T (2013). More *π* electrons make a difference: emergence of many radicals on graphene nanoribbons studied by *ab initio* DMRG theory. J. Chem. Theory Comput..

[21] Gomes J, Mallion R (2001). Aromaticity and ring currents. Chem. Rev..

[22] Yoon ZS, Osuka A, Kim D (2009). Möbius aromaticity and antiaromaticity in expanded porphyrins. Nat. Chem..

[23] Miliordos E (2010). Hückel versus Möbius aromaticity: the particle in a cylinder versus a Möbius strip. Phys. Rev. A.

[24] Wu C-S, Lee P-Y, Chai J-D (2016). Electronic properties of cyclacenes from TAO-DFT. Sci. Rep..

[25] Zimmerman HE (1971). Moebius-hueckel concept in organic chemistry. application of organic molecules and reactions. Acc. Chem. Res..

[26] Heilbronner E (1964). Hückel molecular orbitals of Möbius-type conformations of annulenes. Tetrahedron Lett..

[27] Ajami D, Oeckler O, Simon A, Herges R (2003). Synthesis of a Möbius aromatic hydrocarbon. Nature.

[28] Herges R (2006). Topology in chemistry: designing Möbius molecules. Chem. Rev..

[29] Tanda S (2002). Crystal topology: a Möbius strip of single crystals. Nature.

[30] Schaller GR (2014). Design and synthesis of the first triply twisted Möbius annulene. Nat. Chem..

[31] Chuvilin A (2011). Self-assembly of a sulphur-terminated graphene nanoribbon within a single-walled carbon nanotube. Nat. Mater..

[32] Pavliček N (2017). Synthesis and characterization of triangulene. Nat. Nanotechnol..

[33] Kohn W, Sham LJ (1965). Self-consistent equations including exchange and correlation effects. Phys. Rev..

[34] Xu H-L (2009). Knot-isomers of Möbius cyclacene: how does the number of knots influence the structure and first hyperpolarizability?. J. Phys. Chem. C.

[35] Zhong R-L (2012). Spiral intramolecular charge transfer and large first hyperpolarizability in Möbius cyclacenes: new insight into the localized *π* electrons. ChemPhysChem.

[36] dos Santos MC, Alvarez F (2009). Spin current in the Möbius cyclacene belts. Chem. Phys. Lett..

[37] Cohen AJ, Mori-Sánchez P, Yang W (2008). Insights into current limitations of density functional theory. Science.

[38] Cohen AJ, Mori-Sánchez P, Yang W (2012). Challenges for density functional theory. Chem. Rev..

[39] Gryn’ova G, Coote ML, Corminboeuf C (2015). Theory and practice of uncommon molecular electronic configurations. WIREs Comput. Mol. Sci..

[40] Andersson K, Malmqvist P-Å, Roos BO (1992). Second-order perturbation theory with a complete active space self-consistent field reference function. J. Chem. Phys..

[41] Gidofalvi G, Mazziotti DA (2008). Active-space two-electron reduced-density-matrix method: complete active-space calculations without diagonalization of the *N*-electron hamiltonian. J. Chem. Phys..

[42] Fosso-Tande J, Nguyen T-S, Gidofalvi G, DePrince AE (2016). Large-scale variational two-electron reduced-density-matrix-driven complete active space self-consistent field methods. J. Chem. Theory Comput..

[43] Chai J-D (2012). Density functional theory with fractional orbital occupations. J. Chem. Phys..

[44] Chai J-D (2014). Thermally-assisted-occupation density functional theory with generalized-gradient approximations. J. Chem. Phys..

[45] Chai J-D (2017). Role of exact exchange in thermally-assisted-occupation density functional theory: a proposal of new hybrid schemes. J. Chem. Phys..

[46] Lin C-Y, Hui K, Chung J-H, Chai J-D (2017). Self-consistent determination of the fictitious temperature in thermally-assisted-occupation density functional theory. RSC Adv..

[47] Wu C-S, Chai J-D (2015). Electronic properties of zigzag graphene nanoribbons studied by TAO-DFT. J. Chem. Theory Comput..

[48] Yeh C-N, Chai J-D (2016). Role of Kekulé and non-Kekulé structures in the radical character of alternant polycyclic aromatic hydrocarbons: a TAO-DFT study. Sci. Rep..

[49] Seenithurai S, Chai J-D (2016). Effect of Li adsorption on the electronic and hydrogen storage properties of acenes: a dispersion-corrected TAO-DFT study. Sci. Rep..

[50] Seenithurai S, Chai J-D (2017). Effect of Li termination on the electronic and hydrogen storage properties of linear carbon chains: a TAO-DFT study. Sci. Rep..

[51] Seenithurai S, Chai J-D (2018). Electronic and hydrogen storage properties of Li-terminated linear boron chains studied by TAO-DFT. Sci. Rep..

[52] Yeh C-N, Wu C, Su H, Chai J-D (2018). Electronic properties of the coronene series from thermally-assisted-occupation density functional theory. RSC Adv..

[53] Sadowsky D, McNeill K, Cramer CJ (2010). Electronic structures of [*n*]-cyclacenes (*n* = 6–12) and short, hydrogen-capped, carbon nanotubes. Faraday Discuss..

[54] Pérez-Guardiola A (2018). The role of topology in organic molecules: origin and comparison of the radical character in linear and cyclic oligoacenes and related oligomers. Phys. Chem. Chem. Phys..

[55] Rivero P, Jiménez-Hoyos CA, Scuseria GE (2013). Entanglement and polyradical character of polycyclic aromatic hydrocarbons predicted by projected Hartree-Fock theory. J. Phys. Chem. B.

[56] Shao Y (2015). Advances in molecular quantum chemistry contained in the Q-Chem 4 program package. Mol. Phys..

[57] Dirac PAM (1930). Note on exchange phenomena in the Thomas-Fermi atom. Proc. Cambridge Philos. Soc..

[58] Perdew JP, Wang Y (1992). Accurate and simple analytic representation of the electron-gas correlation energy. Phys. Rev. B.

[59] Chan S-P, Chen G, Gong X, Liu Z-F (2003). Oxidation of carbon nanotubes by singlet O_2_. Phys. Rev. Lett..

[60] Cohen AJ, Tozer DJ, Handy NC (2007). Evaluation of 〈S^2^〉 in density functional theory. J. Chem. Phys..

[61] Gupta S, Saxena A (2011). Geometrical interpretation and curvature distribution in nanocarbons. J. Appl. Phys..

