# Supplementary material for: Electronic Properties of Möbius Cyclacenes Studied by Thermally-Assisted-Occupation Density Functional Theory
Source: Sci Rep. 2019 Feb 27;9:2907. doi: 10.1038/s41598-019-39524-4 (PMC6393452; doi:10.1038/s41598-019-39524-4)
Supplement: Supplementary file 1 — Supplementary Material to: Electronic Properties of Möbius Cyclacenes Studied by Thermally-Assisted-Occupation Density Functional Theory [file 41598_2019_39524_MOESM1_ESM.pdf]

Supplementary Material to: Electronic Properties of Möbius Cyclacenes Studied by Thermally-Assisted-Occupation Density Functional Theory

Jui-Hui Chung[1] and Jeng-Da Chai[1,2,3,*]

[1] *Department of Physics, National Taiwan University, Taipei 10617, Taiwan*

[2] *Center for Theoretical Physics, National Taiwan University, Taipei 10617, Taiwan*

[3] *Center for Quantum Science and Engineering, National Taiwan University, Taipei 10617, Taiwan*

[*] Author to whom correspondence should be addressed. Electronic mail: jdchai@phys.ntu.edu.tw

LIST OF TABLES

S1 Singlet-triplet energy gap (in kcal/mol) of Möbius n-cyclacene/n-cyclacene/n-acene, obtained with spin-unrestricted TAO-LDA. 3

S2 Vertical ionization potential (in eV) for the ground state of Möbius n-cyclacene/n-cyclacene/n-acene, obtained with spin-unrestricted TAO-LDA. . . 6

S3 Vertical electron affinity (in eV) for the ground state of Möbius n-cyclacene/n-cyclacene/n-acene, obtained with spin-unrestricted TAO-LDA. 9

S4 Fundamental gap (in eV) for the ground state of Möbius n-cyclacene/n-cyclacene/n-acene, obtained with spin-unrestricted TAO-LDA. 12

S5 Symmetrized von Neumann entropy for the ground state of Möbius n-cyclacene/n-cyclacene/n-acene, obtained with spin-unrestricted TAO-LDA. . . 16

TABLES

TABLE S1: Singlet-triplet energy gap (in kcal/mol) of Möbius n-cyclacene/n-cyclacene/n-acene, obtained with spin-unrestricted TAO-LDA.

n	Möbius n-cyclacene	n-cyclacene	n-acene
8	5.53	11.14	7.84
9	5.09	2.84	6.66
10	5.13	9.02	5.91
11	4.79	2.49	5.32
12	4.07	6.35	4.82
13	4.00	2.77	4.38
14	3.40	4.28	3.98
15	3.37	3.04	3.65
16	3.05	3.09	3.37
17	2.93	3.06	3.14
18	2.78	2.52	2.94
19	2.60	2.83	2.77
20	2.52	2.25	2.62
21	2.34	2.51	2.49
22	2.28	2.11	2.37
23	2.14	2.21	2.25
24	2.07	2.00	2.15
25	1.97	1.97	2.06
26	1.90	1.89	1.98
27	1.83	1.80	1.90
28	1.76	1.77	1.83
29	1.70	1.67	1.76
30	1.64	1.65	1.70

31	1.59	1.57	1.64
32	1.54	1.54	1.59
33	1.49	1.48	1.54
34	1.45	1.44	1.49
35	1.40	1.40	1.45
36	1.37	1.36	1.40
37	1.33	1.32	1.36
38	1.29	1.28	1.33
39	1.26	1.25	1.29
40	1.23	1.22	1.26
41	1.20	1.19	1.23
42	1.17	1.16	1.20
43	1.14	1.14	1.17
44	1.11	1.11	1.14
45	1.09	1.09	1.11
46	1.07	1.06	1.09
47	1.04	1.04	1.07
48	1.02	1.02	1.04
49	1.00	1.00	1.02
50	0.98	0.98	1.00
51	0.96	0.96	0.98
52	0.94	0.94	0.96
53	0.92	0.92	0.94
54	0.91	0.90	0.92
55	0.89	0.89	0.91
56	0.87	0.87	0.89
57	0.86	0.86	0.87
58	0.84	0.84	0.86
59	0.83	0.83	0.84
60	0.82	0.81	0.83

61	0.80	0.80	0.82
62	0.79	0.79	0.80
63	0.78	0.78	0.79
64	0.76	0.76	0.78
65	0.75	0.75	0.76
66	0.74	0.74	0.75
67	0.73	0.73	0.74
68	0.72	0.72	0.73
69	0.71	0.71	0.72
70	0.70	0.70	0.71
71	0.69	0.69	0.70
72	0.68	0.68	0.69
73	0.67	0.67	0.68
74	0.66	0.66	0.67
75	0.65	0.65	0.66
76	0.64	0.64	0.65
77	0.63	0.63	0.64
78	0.63	0.63	0.64
79	0.62	0.62	0.63
80	0.61	0.61	0.62
81	0.60	0.60	0.61
82	0.60	0.60	0.60
83	0.59	0.59	0.60
84	0.58	0.58	0.59
85	0.57	0.57	0.58
86	0.57	0.57	0.58
87	0.56	0.56	0.57
88	0.56	0.55	0.56
89	0.55	0.55	0.56
90	0.54	0.54	0.55

91	0.54	0.54	0.54
92	0.53	0.53	0.54
93	0.53	0.52	0.53
94	0.52	0.52	0.53
95	0.51	0.51	0.52
96	0.51	0.51	0.51
97	0.50	0.50	0.51
98	0.50	0.50	0.50
99	0.49	0.49	0.50
100	0.49	0.49	0.49

TABLE S2: Vertical ionization potential (in eV) for the ground state of Möbius n-cyclacene/n-cyclacene/n-acene, obtained with spin-unrestricted TAO-LDA.

n	Möbius n-cyclacene	n-cyclacene	n-acene
8	5.40	5.54	5.44
9	5.43	5.30	5.33
10	5.31	5.38	5.23
11	5.24	5.19	5.15
12	5.22	5.22	5.08
13	5.11	5.10	5.01
14	5.11	5.08	4.96
15	5.01	5.02	4.91
16	5.01	4.98	4.86
17	4.94	4.95	4.82
18	4.92	4.90	4.78
19	4.88	4.88	4.75
20	4.85	4.83	4.71
21	4.82	4.81	4.69
22	4.79	4.78	4.66

23	4.77	4.75	4.63
24	4.74	4.73	4.61
25	4.72	4.70	4.59
26	4.69	4.68	4.57
27	4.67	4.66	4.55
28	4.65	4.64	4.53
29	4.63	4.62	4.51
30	4.61	4.60	4.50
31	4.60	4.59	4.48
32	4.58	4.57	4.47
33	4.56	4.55	4.45
34	4.55	4.54	4.44
35	4.53	4.53	4.43
36	4.52	4.51	4.42
37	4.51	4.50	4.40
38	4.49	4.49	4.39
39	4.48	4.47	4.38
40	4.47	4.46	4.37
41	4.46	4.45	4.36
42	4.45	4.44	4.35
43	4.44	4.43	4.35
44	4.43	4.42	4.34
45	4.42	4.41	4.33
46	4.41	4.40	4.32
47	4.40	4.40	4.31
48	4.39	4.39	4.31
49	4.38	4.38	4.30
50	4.38	4.37	4.29
51	4.37	4.36	4.29
52	4.36	4.36	4.28

53	4.35	4.35	4.27
54	4.35	4.34	4.27
55	4.34	4.33	4.26
56	4.33	4.33	4.26
57	4.33	4.32	4.25
58	4.32	4.32	4.25
59	4.31	4.31	4.24
60	4.31	4.30	4.23
61	4.30	4.30	4.23
62	4.30	4.29	4.23
63	4.29	4.29	4.22
64	4.28	4.28	4.22
65	4.28	4.28	4.21
66	4.27	4.27	4.21
67	4.27	4.27	4.20
68	4.26	4.26	4.20
69	4.26	4.26	4.20
70	4.26	4.25	4.19
71	4.25	4.25	4.19
72	4.25	4.24	4.18
73	4.24	4.24	4.18
74	4.24	4.23	4.18
75	4.23	4.23	4.17
76	4.23	4.23	4.17
77	4.23	4.22	4.17
78	4.22	4.22	4.16
79	4.22	4.22	4.16
80	4.21	4.21	4.16
81	4.21	4.21	4.15
82	4.21	4.20	4.15

83	4.20	4.20	4.15
84	4.20	4.20	4.15
85	4.20	4.19	4.14
86	4.19	4.19	4.14
87	4.19	4.19	4.14
88	4.19	4.19	4.14
89	4.19	4.18	4.13
90	4.18	4.18	4.13
91	4.18	4.18	4.13
92	4.18	4.17	4.13
93	4.17	4.17	4.12
94	4.17	4.17	4.12
95	4.17	4.17	4.12
96	4.17	4.16	4.12
97	4.16	4.16	4.11
98	4.16	4.16	4.11
99	4.16	4.15	4.11
100	4.16	4.15	4.11

TABLE S3: Vertical electron affinity (in eV) for the ground state of Möbius n-cyclacene/n-cyclacene/n-acene, obtained with spin-unrestricted TAO-LDA.

n	Möbius n-cyclacene	n-cyclacene	n-acene
8	1.52	1.59	2.06
9	1.81	1.92	2.19
10	1.89	1.91	2.30
11	2.01	2.17	2.39
12	2.19	2.18	2.48
13	2.22	2.35	2.55
14	2.38	2.38	2.61

15	2.39	2.48	2.67
16	2.51	2.54	2.72
17	2.54	2.59	2.77
18	2.61	2.65	2.81
19	2.66	2.69	2.85
20	2.70	2.74	2.89
21	2.75	2.77	2.92
22	2.78	2.82	2.95
23	2.83	2.85	2.98
24	2.85	2.88	3.00
25	2.89	2.91	3.03
26	2.92	2.94	3.05
27	2.94	2.97	3.07
28	2.97	2.99	3.09
29	2.99	3.01	3.11
30	3.02	3.03	3.13
31	3.04	3.06	3.15
32	3.06	3.07	3.16
33	3.08	3.09	3.18
34	3.09	3.11	3.19
35	3.11	3.13	3.21
36	3.13	3.14	3.22
37	3.14	3.16	3.23
38	3.16	3.17	3.25
39	3.17	3.18	3.26
40	3.18	3.20	3.27
41	3.20	3.21	3.28
42	3.21	3.22	3.29
43	3.22	3.23	3.30
44	3.23	3.24	3.31

45	3.24	3.26	3.32
46	3.25	3.27	3.33
47	3.26	3.27	3.33
48	3.27	3.28	3.34
49	3.28	3.29	3.35
50	3.29	3.30	3.36
51	3.30	3.31	3.36
52	3.31	3.32	3.37
53	3.32	3.33	3.38
54	3.33	3.33	3.39
55	3.33	3.34	3.39
56	3.34	3.35	3.40
57	3.35	3.35	3.40
58	3.36	3.36	3.41
59	3.36	3.37	3.42
60	3.37	3.37	3.42
61	3.37	3.38	3.43
62	3.38	3.39	3.43
63	3.39	3.39	3.44
64	3.39	3.40	3.44
65	3.40	3.40	3.45
66	3.40	3.41	3.45
67	3.41	3.41	3.46
68	3.41	3.42	3.46
69	3.42	3.42	3.46
70	3.42	3.43	3.47
71	3.43	3.43	3.47
72	3.43	3.44	3.48
73	3.44	3.44	3.48
74	3.44	3.45	3.48

75	3.45	3.45	3.49
76	3.45	3.46	3.49
77	3.46	3.46	3.50
78	3.46	3.46	3.50
79	3.46	3.47	3.50
80	3.47	3.47	3.51
81	3.47	3.48	3.51
82	3.48	3.48	3.51
83	3.48	3.48	3.52
84	3.48	3.49	3.52
85	3.49	3.49	3.52
86	3.49	3.49	3.52
87	3.49	3.50	3.53
88	3.50	3.50	3.53
89	3.50	3.50	3.53
90	3.50	3.51	3.54
91	3.51	3.51	3.54
92	3.51	3.51	3.54
93	3.51	3.52	3.54
94	3.51	3.52	3.55
95	3.52	3.52	3.55
96	3.52	3.52	3.55
97	3.52	3.53	3.55
98	3.53	3.53	3.56
99	3.53	3.53	3.56
100	3.53	3.53	3.56

TABLE S4: Fundamental gap (in eV) for the ground state of Möbius n-cyclacene/n-cyclacene/n-acene, obtained with spin-unrestricted TAO-LDA.

n	Möbius n-cyclacene	n-cyclacene	n-acene
8	3.88	3.95	3.38
9	3.63	3.38	3.13
10	3.41	3.47	2.93
11	3.22	3.02	2.76
12	3.03	3.04	2.60
13	2.89	2.76	2.46
14	2.73	2.70	2.34
15	2.62	2.54	2.23
16	2.50	2.45	2.13
17	2.40	2.36	2.05
18	2.31	2.25	1.97
19	2.22	2.19	1.89
20	2.15	2.09	1.83
21	2.07	2.04	1.76
22	2.01	1.96	1.71
23	1.94	1.91	1.65
24	1.88	1.84	1.60
25	1.83	1.79	1.56
26	1.78	1.74	1.52
27	1.73	1.69	1.47
28	1.68	1.65	1.44
29	1.64	1.61	1.40
30	1.60	1.57	1.37
31	1.56	1.53	1.33
32	1.52	1.50	1.30
33	1.49	1.46	1.27
34	1.45	1.43	1.25
35	1.42	1.40	1.22
36	1.39	1.37	1.20

37	1.36	1.34	1.17
38	1.34	1.32	1.15
39	1.31	1.29	1.13
40	1.29	1.27	1.11
41	1.26	1.24	1.09
42	1.24	1.22	1.07
43	1.22	1.20	1.05
44	1.20	1.18	1.03
45	1.17	1.16	1.01
46	1.16	1.14	1.00
47	1.14	1.12	0.98
48	1.12	1.11	0.96
49	1.10	1.09	0.95
50	1.08	1.07	0.94
51	1.07	1.05	0.92
52	1.05	1.04	0.91
53	1.03	1.02	0.89
54	1.02	1.01	0.88
55	1.01	0.99	0.87
56	0.99	0.98	0.86
57	0.98	0.97	0.85
58	0.96	0.95	0.84
59	0.95	0.94	0.82
60	0.94	0.93	0.81
61	0.93	0.92	0.80
62	0.91	0.91	0.79
63	0.90	0.89	0.78
64	0.89	0.88	0.77
65	0.88	0.87	0.77
66	0.87	0.86	0.76

67	0.86	0.85	0.75
68	0.85	0.84	0.74
69	0.84	0.83	0.73
70	0.83	0.82	0.72
71	0.82	0.81	0.72
72	0.81	0.80	0.71
73	0.80	0.80	0.70
74	0.80	0.79	0.69
75	0.79	0.78	0.69
76	0.78	0.77	0.68
77	0.77	0.76	0.67
78	0.76	0.75	0.67
79	0.75	0.75	0.66
80	0.75	0.74	0.65
81	0.74	0.73	0.65
82	0.73	0.72	0.64
83	0.73	0.72	0.63
84	0.72	0.71	0.63
85	0.71	0.70	0.62
86	0.70	0.70	0.62
87	0.70	0.69	0.61
88	0.69	0.69	0.61
89	0.69	0.68	0.60
90	0.68	0.67	0.59
91	0.67	0.67	0.59
92	0.67	0.66	0.58
93	0.66	0.66	0.58
94	0.66	0.65	0.58
95	0.65	0.64	0.57
96	0.65	0.64	0.57

97	0.64	0.63	0.56
98	0.63	0.63	0.56
99	0.63	0.62	0.55
100	0.62	0.62	0.55

TABLE S5: Symmetrized von Neumann entropy for the ground state of Möbius n-cyclacene/n-cyclacene/n-acene, obtained with spin-unrestricted TAO-LDA.

n	Möbius n-cyclacene	n-cyclacene	n-acene
8	1.68	0.99	1.34
9	1.85	2.50	1.52
10	1.90	1.23	1.67
11	2.02	2.80	1.82
12	2.33	1.74	1.98
13	2.41	2.83	2.16
14	2.74	2.42	2.36
15	2.82	2.90	2.55
16	3.07	3.06	2.75
17	3.21	3.10	2.94
18	3.38	3.55	3.13
19	3.60	3.43	3.31
20	3.74	3.92	3.50
21	3.98	3.85	3.69
22	4.12	4.24	3.88
23	4.35	4.29	4.07
24	4.51	4.55	4.26
25	4.71	4.72	4.44
26	4.90	4.90	4.63
27	5.08	5.11	4.82
28	5.28	5.26	5.01

29	5.46	5.49	5.20
30	5.66	5.64	5.39
31	5.84	5.86	5.58
32	6.04	6.03	5.77
33	6.22	6.23	5.95
34	6.41	6.41	6.14
35	6.60	6.60	6.33
36	6.79	6.79	6.52
37	6.98	6.98	6.71
38	7.17	7.17	6.90
39	7.36	7.36	7.09
40	7.54	7.55	7.27
41	7.73	7.73	7.46
42	7.92	7.93	7.65
43	8.11	8.11	7.84
44	8.30	8.30	8.03
45	8.49	8.49	8.22
46	8.68	8.68	8.41
47	8.86	8.87	8.59
48	9.05	9.06	8.78
49	9.24	9.24	8.97
50	9.43	9.43	9.16
51	9.62	9.62	9.35
52	9.81	9.81	9.54
53	10.00	10.00	9.73
54	10.19	10.19	9.92
55	10.37	10.38	10.10
56	10.56	10.57	10.29
57	10.75	10.75	10.48
58	10.94	10.94	10.67

59	11.13	11.13	10.86
60	11.32	11.32	11.05
61	11.51	11.51	11.23
62	11.70	11.70	11.42
63	11.88	11.89	11.61
64	12.07	12.07	11.80
65	12.26	12.26	11.99
66	12.45	12.45	12.18
67	12.64	12.64	12.37
68	12.83	12.83	12.56
69	13.02	13.02	12.74
70	13.20	13.21	12.93
71	13.39	13.39	13.12
72	13.58	13.58	13.31
73	13.77	13.77	13.50
74	13.96	13.96	13.69
75	14.15	14.15	13.88
76	14.34	14.34	14.06
77	14.53	14.53	14.25
78	14.71	14.71	14.44
79	14.90	14.90	14.63
80	15.09	15.09	14.82
81	15.28	15.28	15.01
82	15.47	15.47	15.20
83	15.66	15.66	15.38
84	15.85	15.85	15.57
85	16.03	16.04	15.76
86	16.22	16.22	15.95
87	16.41	16.41	16.14
88	16.60	16.60	16.33

89	16.79	16.79	16.52
90	16.98	16.98	16.70
91	17.17	17.17	16.89
92	17.36	17.36	17.08
93	17.54	17.54	17.27
94	17.73	17.73	17.46
95	17.92	17.92	17.65
96	18.11	18.11	17.84
97	18.30	18.30	18.02
98	18.49	18.49	18.21
99	18.68	18.68	18.40
100	18.86	18.86	18.59
